# Effectiveness of online expressive writing in reducing psychological distress among the asymptomatic COVID-19 patients in Fangcang Hospitals: A quasi-experiment study

**DOI:** 10.3389/fpsyg.2022.1042274

**Published:** 2023-01-04

**Authors:** Xican Zheng, Jingrui Qu, Jun Xie, Wei Yue, Xuejun Liang, Zhen Shi, Jing Bai, Zhiyan Sun, Fangna Cheng, Xiaoxia Li, Chunxia Liu

**Affiliations:** ^1^Administration Office of the 988th Hospital, Zhengzhou, China; ^2^Department of Psychiatry, the 988th Hospital, Zhengzhou, China; ^3^Department of Ophthalmology, the 988th Hospital, Zhengzhou, China; ^4^Department of Aviators Healthcare, the 988th Hospital, Zhengzhou, China; ^5^Department of Obstetrics and Gynecology, the 988th Hospital, Zhengzhou, China; ^6^Department of Rehabilitation, the 988th Hospital, Zhengzhou, China

**Keywords:** COVID-19, asymptomatic, Fangcang Hospitals, expressive writing, psychological distress

## Abstract

**Objective:**

This study aimed to assess the applicability and effectiveness of an online format of expressive writing (EW) in reducing psychological distress among the asymptomatic COVID-19 patients in Fangcang Hospitals with a quasi-experiment.

**Method:**

Altogether 244 patients were assigned to the EW group(n=122) and the control group(n=122). Besides the routine psychological intervention (broadcast relaxation training at a fixed time) in Fangcang hospitals, The EW group was engaged in 8-day theme-based adaption EW intervention, whereas the control group received no interventions. All the participants were tested with the Brief Profile of Mood States (BPOMS) and Inpatient Mental Health Preliminary Screening Scale(IMHPS) before and after the intervention. After the intervention, the writing quality and intervention satisfaction of the EW group were evaluated by a self-designed writing quality questionnaire and EW satisfaction questionnaire.

**Results:**

The results indicated that the EW significantly improved in the BPOMS test, whereas the control group showed no significant change. The IMHPS score in the control group was statistically deteriorated than that before intervention, whereas the EW group showed no significant change. The writing quality was highly correlated with the score change of BPMOS. The overall satisfaction of patients with EW was 81.13%.

**Conclusion:**

EW can reduce psychological distress among the asymptomatic COVID-19 patients in Fangcang Hospitals. The higher the quality of writing, the greater the improvement of mood states. As a new form of psychological intervention in Fangcang hospitals with high patient satisfaction, EW has a value of popularization and application.

## Introduction

An outbreak of COVID-19 caused by the Omicron variant occurred in Shanghai in 2022. Investigation shows that asymptomatic cases account for 90% of the infected cases. To stop the spread of the virus, Shanghai has opened a number of Fangcang hospitals to receive asymptomatic patients. Fangcang hospitals refer to a novel concept: large, temporary hospitals built by converting public venues, such as stadiums and exhibition centers, into healthcare facilities to isolate patients with mild to moderate symptoms of an infectious disease from their families and communities, while providing medical care, disease monitoring, food, shelter, and social activities (Chen et al., 2020). While Fangcang hospitals have many advantages in controlling the spread of the epidemic, the relatively open space may make patients lack privacy, and the combination of many factors such as isolation from family and friends, the uncertainty of disease outcome may aggravate the patients’ stress, anxiety, depression, and other adverse mood states. According to the Shanghai International Convention and Exhibition Center, the average length of hospital stay was 7.18 days. Most patients can be discharged within about 7 days of hospitalization. But, if some patients have not reached the discharge standard for more than 7 days, they are faced with greater physical and mental threats. Especially some asymptomatic patients may develop into confirmed COVID-19 patients with clinical symptoms, which causes may elicit fear and irrational responses. A systematic review and meta-analysis on the psychological impact of COVID-19 revealed increased rates of depression and anxiety across health care workers, patients, and the general public (Luo et al., 2020). One-fifth of asymptomatic or mildly symptomatic patients with COVID-19 had anxiety and/or depression (Jeong et al., 2020). Previous studies have shown that patients in Fangcang hospitals, apart from their familiar living environment, are full of loneliness and have different degrees of psychological problems (Fu et al., 2022). However, due to the isolated nature of Fangcang hospitals and the limitation of psychological professional resources, the application of traditional, face-to-face psychological intervention is facing challenge. There is a need for novel approaches, strategies, and interventions that apply to a large number of people to reduce the short and long-term adverse psychological effects of the pandemic (Markovic et al., 2020).

### Expressive writing

In recent years, Expressive writing (EW) has been found to have beneficial effects on physical and mental health (Nicholls, 2009). EW is an intervention in which one is asked to disclose one’s deepest thoughts and feelings surrounding a stressful life event, initially introduced by Pennebaker in 1986 (Pennebaker and Chung, 2011; Andersson and Conley, 2013). It is also a “stand-alone” technique for treating depressive, anxious, and posttraumatic stress disorder symptoms (Graf et al., 2008; Reinhold et al., 2018). Besides the assumed beneficial health effects, the parsimony of writing treatments and the considerable potential to close gaps in the provision of treatment through remote (e.g., online) delivery may have contributed to the treatment’s continuing popularity over the last three decades. However, the results were inconclusive at the meta-analytic level. The largest and the most inclusive meta-analysis to date (Frattaroli, 2006) found a significant positive overall effect of EW. More specifically, he found a significant average impact for reducing symptoms of depression, as well as for distress and anxiety. On the contrary, several meta-analyses have been conducted that showed no or only minor sized beneficial effects of the original EW assignments in improving mental health (Frisina et al., 2004; Meads and Nouwen, 2005; Mogk et al., 2006; Reinhold et al., 2018; Pavlacic et al., 2019).

Thus, many conditions were examined to determine if there were optimal settings and processes for EW to produce a benefit. Some variables were determined to be important. For instance, a larger number of writing sessions, longer writing periods, and more detailed disclosure appeared to enhance the benefit of EW interventions (Smyth et al., 2008). Furthermore, to better understand processes of change in response to writing, many researchers have attempted to examine the writing content. These studies have shown that moderate amounts of negative emotion words and increases in causal/insight and positive emotion words as the sessions progress are related to improved health outcomes. Although the traditional EW instructions ask participants to focus on negative events, the improved outcome has also been associated with topics that ask for focus on positive aspects of such events (e.g., future positive goals; King and Miner, 2000). Stanton et al. (2002) found that asking patients with breast cancer to explore the potential benefit of their stressful medical experiences through writing led to reductions in both physical symptoms and symptom-related medical visits. Modifying the standard writing instructions to direct written narratives toward “intensely positive experiences” produced health benefits.

Based on these results, future research studies should focus on these and related characteristics to identify and develop the most effective conditions for administering a structured writing intervention. These findings motivated adaptions of the original paradigm to increase writing treatments observed beneficial treatment effects (Smyth and Pennebaker, 2008).

### Promises of EW in the context of COVID-19 pandemic

Researchers have explored the EW intervention in the Context of the COVID-19 Pandemic in the general population and healthcare workers. Some studies reported that EW intervention improved resilience and buffered the negative side effects of stress during the COVID-19 pandemic (Bechard et al., 2021; Marschin and Herbert, 2021; Procaccia et al., 2021). While another study (Markovic et al., 2020) shows that when applied in the context of the COVID-19 pandemic, it did not benefit one’s mental health. To date, the effectiveness of EW intervention among asymptomatic COVID-19 patients is less known. Regarding accessibility, the EW protocol is ideally suited for use with patients in quarantine and observing social distancing. EW could provide a valuable tool to promote mental health with minimal contact with a therapist (Gerger et al., 2021). Furthermore, Chinese people are reserved in their emotions express. In Fangcang hospital, EW may be beneficial for individuals with limited availability of emotional outlets in their social environment.

### Current study

This study aimed to assess the effectiveness of EW interventions in reducing the psychological distress of the asymptomatic COVID-19 patients in Fangcang Hospitals by conducting a quasi-experiment. Besides, the correlation between writing quality and psychological change quantity was been explored. Finally, satisfaction with EW interventions among participants was been assessed. Based on previous research conclusions, for persons in a state of crisis or with high levels of distress, clinicians might be concerned that EW could exacerbate harm (Lepore and Smyth, 2002). In this study, we modified the standard writing instructions to direct written narratives toward “intensely positive experiences.” Our programs combined the original paradigm and positive writing paradigm (Baikie et al., 2012), which not only instructed participants to“really let go and experience the emotion associated with the stressor,” but also focus on the perceived benefits of the stressor to “look on the bright side” or to find meaning in the event. In such instances, this alternative approach, which enables a person to adjust by focusing on positive aspects of a stressor, might be most beneficial. We hypothesized that receiving the EW intervention would be effective in the reduction of psychological distress. We may find more evident results in this more focused population to provide valuable references and scientific evidence for the application of EW interventions.

## Materials and methods

### Study design and participants

In the present study, convenience sampling was used to select asymptomatic patients admitted to Hall 5.2, Branch 6, The Fangcang Hospital, which was reconstructed based on Shanghai International Convention and Exhibition Center. Fangcang hospitals are open spaces, and the ward beds are close to each other. In order to reduce the contamination between the two groups, A non-randomized quasi-experimental study design was used. To be eligible for the study, participants had to be aged between 18 and 60 years (Because online EW has a time limit, patients over 60 years may not use mobile phones expertly leading to some impacts on the result); stayed in the hospital for 7 days and still did not meet the discharge criteria; no mental illness; and junior high school education or above. Patients concomitant with other underlying diseases (e.g., hypertension, diabetes, coronary heart disease, and tumor) were excluded. The patients whose hospitalization stay is the 7th day will be collected from the information system of the Fangcang hospitals from May 1 to May 10, 2022. Eligible participants who decided to participate were then asked to sign an informed consent form. The collected patients who belong to zone E are the EW group, and those who belong to zone D are the control group. This study was approved by the Medical Ethics Committee of the 988th Hospital (Protocol number #2022-GZFC652002).

The formula of *N*= ${\left[\frac{2\left(\mu_{\alpha }+\mu_{\beta }\right)\sigma }{\delta }\right]}^{2}$ was applied to calculate the sample size. Set the type *I* error of hypothesis testing as 0.05, the type *II* error of hypothesis testing as 0.1, and the sample size ratio of the EW group and the control group as 1. By checking the critical value table, it was found that the $\mu_{\alpha }$ is 1.96, and the $\mu_{\beta }$ is 1.28. The allowable error δ is 15, which calculated according to the change of mood states of the two groups in the pre-experiment. The change value of the overall standard deviation σ is 16.53. We figured out that the sample size of each group should be at least 51.

### Procedure

Patients in EW groups wrote according to an adaption instruction under the guidance of the researchers. They were instructed to write without regard to spelling, style, or grammar and were informed that their written narratives would remain confidential. Writing takes place for 8 days, 15–30 min daily. The researchers gave immediate responses to questions raised by the participants in time. The control group just completed the pre-intervention and post-intervention measures at the same time. We did not use the usual control group assignment of writing about superficial topics, such as how they use their time, because patients in the control group might refuse to comply or drop out of the study. Participants in both groups received routine psychological intervention in Fangcang hospitals (broadcast relaxation training at a fixed time).

In the pre-session, (a) Set up an intervention team: the intervention team was composed of 5 nurses who obtained the qualification of psychological consultant and a graduate student in Applied psychology who systematically studied EW. (b) Group training: The graduate student trained group members on the principles of EW, implementation methods, and scale assessment. (c) Protocol determination: Group members jointly determined the EW plan for asymptomatic patients according to professional and situational perspectives. The specific content was shown in Table 1. (d) Division of labor: Among the intervention team members, nurse 1 was responsible for the EW intervention, nurse 2 was responsible for the evaluation of the writing quality of patients, nurses 3, 4, and 5 were responsible for the scale assessment, and the graduate students were responsible for the statistical analysis of data.

**Table 1:** EW intervention programs.

Stage	Topic	Aim	Instructions
First stage (first and second day)	emotion perception	To help patients feel the thoughts and emotion	(1) What thoughts and emotions do you feel when the test result fails to meet the discharge standard?
			(2) Please write down how these thoughts and emotions have affected your life (in terms of relationships, work, etc.).
Second stage (the 3th-4th day)	cognitive appraisal	To help patients integrate and reorganize cognition to find positive experiences brought by the disease	(1) Please describe and evaluate your knowledge of the asymptomatic infections with COVID-19;
			(2) What kind of help have you received from your family, friends, or social organizations since the nucleic acid testing was positive? How do you feel? (3) Did this hospitalization bring you positive experiences (e.g., relationship with family, change in attitude, personal growth, etc.)?
The third stage (the 5th-6th day)	unlock potential	To help patients recall ways to cope with setbacks and regain their confidence.	(1) How long is the longest time to be sick before? How did you deal with this situation?
			(2) How did you successfully deal with negative emotions or the most significant setbacks in the past?
The fourth stage (the 7th-8th day)	look ahead	To help patients feel the impact of the intervention and make plans for the future.	(1) What have you gained from this intervention? How has your thinking changed?
			(2) How do you plan to work and live after discharge?

In the implementation session, Patients were asked into the WeChat group by scanning the QR code. Patients on May 1 were enrolled in EW group 1, and patients on May 2 were enrolled in EW group 2. In this order, 10 groups were generated. Each EW group started to begin EW on the second day. All 10 groups were managed by nurse 1, responsible for introducing the role, methods, and precautions to patients, especially pledging the confidentiality of their writing content. Avoid the period of patients’ treatment; the EW time is 19:00 to 19:30 every day. Nurse 1 sent the instruction to the EW group at 19:00, and the patient began to write according to the guide. If patients need to deal with other matters during this period, it can be postponed to 22:00 on the same day. Writing is not limited to the number of words, but the prescribed time, that is, at least 20 min for consecutive writing; each stage of the topic can be written in 2 days. Patients wrote through the mobile phone and sent the writing content to Nurse 2.

In the post-session, nurse 2 was responsible for evaluating the writing quality and feedback to the participants. The nurse only commends that the patients finished writing intraday and did not evaluate the writing content to avoid confusing the intervention effect. Other team members did not participate in the process to ensure the unity of evaluation standards. The quality of writing was evaluated and recorded according to the following criteria: (a) the length of the writing (0 points for less than 50 words, 1 point for 50 to 100 words, 2 points for 101 to 150 words, 3 points for 151 to 200 words, 4 points for 201 to 205 words, and 5 points for more than 250 words; b) How well does the writing fit the topic? (c)To what extent does the writing describe a positive emotional experience? Each item is scored on a 6-point ranging from “not at all “(0 points) to “a great deal” (5 points); the total score ranges from 0 to 15.

### Measure

The two groups were given a battery of tests online 1 day before intervention and 1 day after the final session. The severity of psychological distress was assessed using Brief Profile of Mood States (BPOMS). This scale is derived from the Profile of Mood States (POMS) compiled by McNair (McNair, 1992). The concise scale, revised and simplified by Chinese scholars (Chi and Lin, 2003), has good reliability and validity. Cronbach’s α ranges from 0.67 to 0.93. It was used to assess both specific and general mood. The BPOMS is a 30 contains item Likert format (0 = “not at all” and “4 = extremely) questionnaire that measures the specific mood factors of tension (T), depression (D), anger (A), vigor (V), fatigue (F), and confusion (C). A widely used higher-order measure of total mood disturbance (TMD) was assessed by adding the negative mood variables and subtracting the positive variable of vigor. TMD = T + A + F + C + D-V, ranging from-24 to 76 points. Compared with some single emotional assessment tools (such as SDS and SAS), this scale can detect a variety of mood states, is widely used to assess the degree of symptom changes after patients receive specific treatment, and has been proved to have high sensitivity (Wang et al., 2000).

In addition to using self-report questionnaires, we used an observe-rating scale to improve the reliability of psychological state assessment results. This study was authorized to use the Inpatient Mental Health Preliminary Screening Scale (IMHPS), developed by the Nursing Psychology Committee of the Chinese Psychological Society. The scale applies to inpatients in addition to the psychiatry department. Nurses complete the assessment based on observation and brief communication with patients. The scale has 20 items, reflecting the intensity of patients’ emotional responses. Points are scored on a 5-point scale ranging from “never” (0 points) to “always” (4 points). The higher the score, the higher the intensity of the negative emotional response.

Finally, after the intervention, participants from the EW group were asked about their experiences related to the EW intervention: (1) To what extent do you think EW is suitable for asymptomatic infected persons in Fangcang hospitals? (2) To what extent do you think EW plays a role in regulating emotions? EW satisfaction questionnaire is scored on a 6-point scale ranging from “not at all “(0 points) to “a great deal” (5 points), ranging from 0 to 10. The higher the score, the more satisfied the EW intervention.

### Data collection and quality control

Three intervention team members, Nurse 3, Nurse 4, and Nurse 5, who were trained in the assessment of the scales, were responsible for data collection. One day before and 1 day after, nurse 3 sent the BPMOS to the WeChat of the two groups *via* the WeChat-based survey program Questionnaire Star. After the intervention, the EW satisfaction questionnaire was sent to the WeChat of the EW group. According to the list of all included patients, nurses 4 and 5 jointly evaluated patients with the IMHPS 1 day before and 1 day after the intervention under the condition of unknown grouping. In this study, the intervention instructor, writing quality evaluators, and data collectors were separated to reduce subjectivity bias.

### Statistical analyses

SPSS 24.0 statistical software was used for analysis. The data were tested for normality and homogeneity of variance. The Chi-square test and Fisher test compared the demographics of the two groups. Data were summarized using mean ± SD for continuous variables and frequency percentages for categorical variables. The primary outcomes of interest included mood states (BPMOS)and mental health (IMHPS) scores. These measurements were evaluated at baseline and 8 days later. For each group, the mean change was assessed using the 2-Sample t-Test. The difference for each outcome of interest was compared between groups also using the 2-Sample t-Test. Pearson correlation test was used to compare the pairwise correlation between changes in mood states and writing quality. In all cases, *p* < 0.05 was considered statistically significant, and findings were summarized using point estimates and corresponding 95% confidence intervals. Analysis was restricted to participants who completed the study.

## Results

**Figure 1:**
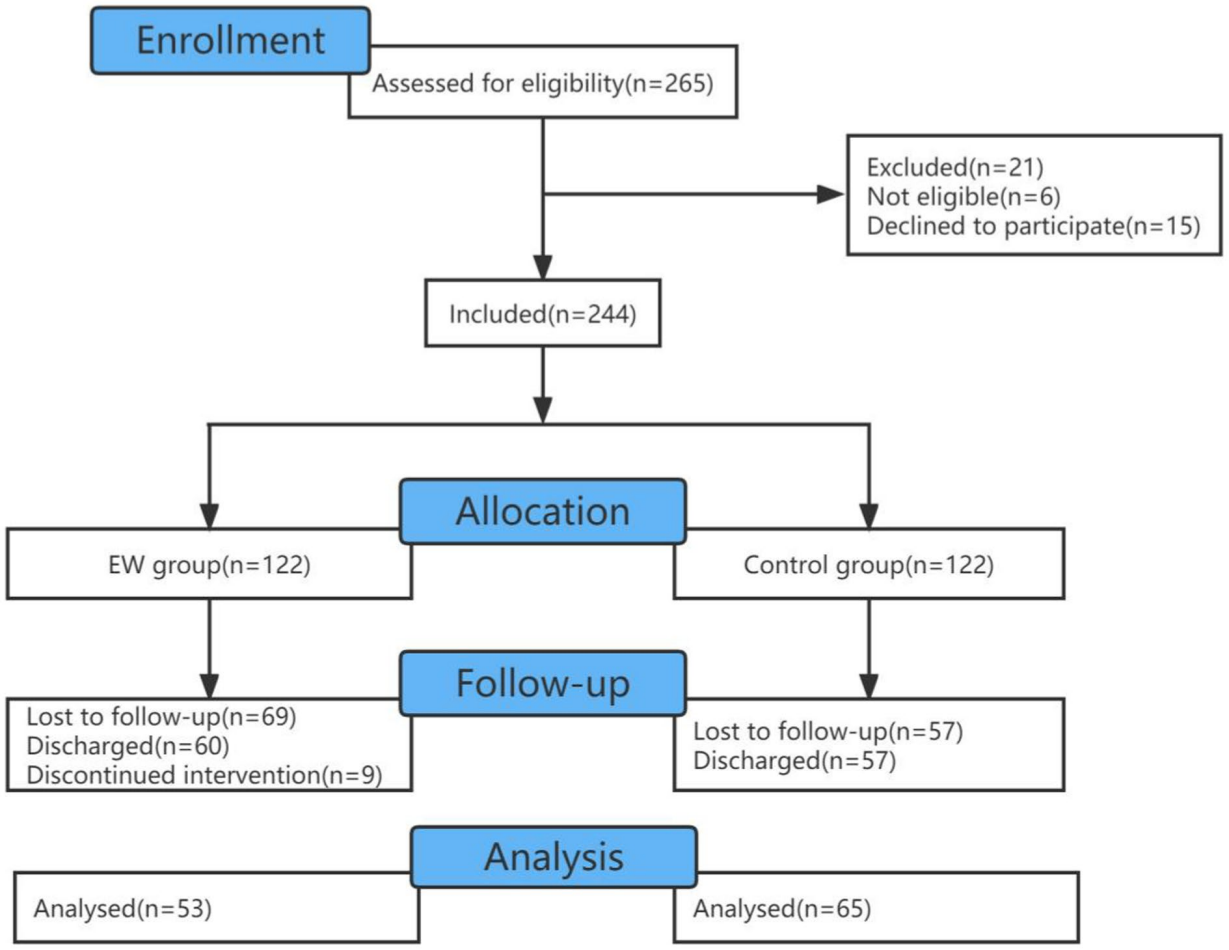
Flow diagram to indicate the included and excluded participants.

During the recruitment period (1 to May 10), 244 patients were eligible and consented to participate in the study (see Figure 1). One hundred seventeen patients were discharged from hospitals during the study, with 60 from the EW group and 57 from the control group. Nine patients dropped out due to incomplete eight times EW. There were 53 cases in the EW group and 65 cases in the control group, totaling 118 participants completing the study. Demographic characteristics were balanced between the EW and control groups (Table 2). All demographic and COVID-related information about the sample is presented in Table 2.

**Table 2:** Comparison of general information between two groups (case).

Variable		EW group (*n* = 53)	Control group (*n* = 65)	$\chi^{2}$	*p*
Gender, *n* (%)	Male	36 (67.9)	38 (58.5)	1.118	0.290
	Female	17 (32.1)	27 (41.5)		
Age, *n* (%)	18 ~ 29	15 (28.3)	24 (36.9)	1.02	0.796
	30 ~ 39	19 (35.8)	21 (32.3)		
	40 ~ 49	9 (17.0)	10 (15.4)		
	50 ~ 60	10 (18.9)	10 (15.4)		
Ethnicity, *n* (%)	Han	53 (100)	62 (95.3)	-	0.251
	Minority	0	3 (4.7)		
Marital status, *n* (%)	Single	14 (26.4)	23 (35.4)	1.401	0.496
	Married	36 (67.9)	40 (61.5)		
	Divorced/widowed	3 (5.7)	2 (3.1)		
Education, *n* (%)	Junior high	15 (28.3)	23 (35.4)	6.494	0.165
	High school	17 (32.1)	20 (30.8)		
	Bachelor	18 (34.0)	21 (32.3)		
	Master and above	3 (5.6)	1 (1.5)		

- is Fisher test

2-Sample t-Test results show that the score of TMD, tension, anger, depression + confusion, and fatigue in the EW group were lower than those in the control group, and the score of vigor was higher than those in the control group after intervention. The differences were statistically significant (*p* < 0.05). The change of the TMD score in the EW group was lower than before intervention, and the vigor score was higher than before intervention. The difference was statistically significant (*p* < 0.05). See Table 3.

**Table 3:** Comparison of BPOMS before and after intervention between two groups (score).

group	Pre-intervention	Post-intervention	*t*	*p*	Pre-intervention	Post-intervention	*t*	*p*
	TMD				tension			
EW group	1.6 ± 13.66	−3.25 ± 7.61	2.257	0.027^*^	1.92 ± 1.86	1.3 ± 1.31	1.986	0.050
Control group	3.51 ± 16.24	7.17 ± 17.65	−1.230	0.221	2.03 ± 2.51	2.51 ± 2.74	−1.032	0.304
***t***	0.679	4.291			0.255	3.131		
***p***	0.498	0.000^***^			0.799	0.002^**^		
	anger				depression + confusion			
EW group	2.34 ± 3.04	1.91 ± 2.34	0.822	0.413	4.75 ± 4.96	3.89 ± 2.79	1.108	0.271
Control group	2.6 ± 3.86	3.2 ± 4.37	−0.829	0.409	5.35 ± 6.13	6.43 ± 6.73	−0.953	0.343
***t***	0.4	2.050			0.574	2.765		
***p***	0.69	0.043^*^			0.567	0.007^**^		
	fatigue				vigor			
EW group	2.47 ± 3.04	1.92 ± 1.63	1.153	0.252	9.89 ± 5.37	12.26 ± 3.00	2.811	0.006^**^
Control group	2.78 ± 3.53	3.55 ± 4.07	−1.15	0.252	9.26 ± 4.98	8.52 ± 4.78	−0.862	0.390
***t***	0.509	2.945			0.654	5.177		
***p***	0.612	0.004^**^			0.514	0.000^***^		

*p < 0.05, **p < 0.01, ***p < 0.001.

After the intervention, the score of IMHPS in the EW group was lower than that in the control group, and the difference was statistically significant (*p* < 0.001). The score in the EW group after intervention was lower than before. Still, the difference was not statistically significant (*p* > 0.05), while the score in the control group after intervention was higher than that before intervention, and the difference was statistically significant (*p* < 0.01). See Table 4.

**Table 4:** Comparison of IMHPS before and after intervention between two groups (score).

Group	IMHPS		*t*	*p*
	Pre-intervention	Post-intervention		
EW group	15.45 ± 8.75	15.13 ± 8.53	0.191	0.849
Control group	16.6 ± 10.70	23.23 ± 13.16	−3.151	0.002^**^
***t***	−0.628	−4.030		
***p***	0.532	0.000^***^		

**p < 0.01, ***p < 0.001.

Since there was little difference in the score of the IMHPS before and after the intervention in the EW group, only the correlation between the change of BPOMS score and writing quality was analyzed. Mood states change = pre-scale score - post-scale score. Those with mood states change greater than 0 (i.e., those with effective EW intervention) were screened out, and the correlation between the improvement effect of mood states and writing quality was analyzed. The results are shown in Table 5. The total score of writing quality, writing length, and the degree of positive emotional experience described by writing was highly correlated with the change of mood states (*r* > 0.8), and the degree of writing fit the topic was low correlated with the change of mood states (0.3 ≤ R < 0.5).

**Table 5:** Correlation analysis between writing quality and score change of BPMOS (*r*).

Item	The length of the writing	How well does the writing fit the topic?	To what extent does the writing describe a positive emotional experience?	The total score of writing quality
Amount of mood states change	0.847^**^	0.430^*^	0.858^**^	0.832^**^

*p < 0.05, **p < 0.01, ***p < 0.001.

The percentage of satisfaction index with medium level and above ranged from 77.36 to 90.57%, as shown in Table 6.

**Table 6:** Descriptive analysis of patients’ satisfaction with EW (score).

Item	*M*	*SD*	Percentage of items with a score of medium level or above (%)
To what extent do you think EW is suitable for asymptomatic infected patients in Fangcang hospitals?	3.38	1.274	77.36
To what extent do you think EW plays a role in reducing psychological distress?	3.83	0.914	90.57
Total scores	7.21	1.801	81.13

## Discussion

The results of BPMOS showed that the TMD score and scores of each dimension of BPOMS in the EW group were significantly better than those in the control group, indicating that EW can reduce the psychological distress of the asymptomatic COVID-19 patients in Fangcang Hospitals. While the EW group still had a certain amount of tension and fatigue after the intervention, the vigor score was significantly improved compared with those before the intervention. It suggests that EW can provide an outlet to express the inhibited content and thus reduce internal tension. Furthermore, thorough the Topic orientation: focusing on the good, guides patients to increase their benefit findings from the illness. For example, some patients noted, “Maybe my self-protection measures are not good enough to make the virus like me so much. It could also be that I had been staying up too late, and my immune system had dropped. This illness may be a reminder to my body and a gift. Whatever you do, you should have a healthy body. Just thinking about it, I feel lucky.” “Although the pandemic brought us many negative experiences, it also made us realize the value of time and the seriousness of our habits.” This result supports the view that stressful events’ negative and positive effects on individuals can exist independently (Fontana and Rosenheck, 1998). In the control group, the negative mood worsened with the increase of the length of hospital stay. It is also consistent with the theory of emotional inhibition (Sloan, 2004) that “the inhibition of emotions associated with stress has a negative impact on health.” Patients who fail to discharge after an average stay (7 days) are prone to have a strong psychological gap when they see their fellow patients discharged successively. They worry about the potential risks caused by newly admitted patients. The control group did not have a channel to reduce the cognitive confusion surrounding an emotional event, which may further aggravate the negative emotions such as tension, anger, and confusion. This study is based on the healing effect of EW (expression and connection, reflection and reinterpretation, acceptance and completion) to design four topics (emotion expression, cognitive appraisal, unlock potential, and look ahead). The results indicated that the modified instructions were ideal for fostering both catharsis and reappraisal. During the upheaval of COVID-19 restrictions, EW can promote individual growth and rebuild their confidence by reducing avoidance of stressors, facilitating problem-solving, and increasing reflection upon epidemic and holistic life balance.

The IMHPS results showed that EW could prevent the aggravation of negative emotions among patients in Fangcang hospitals. In order to ensure the objectivity and comprehensiveness of the assessment results, the observe-rating scale was used. The analysis results showed that the IMHPS and BPOMS scores of inpatients were almost consistent; that is, the psychological state of the EW group was significantly better than that of the control group after the intervention, and the psychological state of the EW group was improved compared with that before the intervention. The only difference is that the psychological state of the control group was significantly aggravated after 8 days (*p* < 0.01) in the IMHPS results. The possible reason is that patients in the control group fail to vent and transform their emotions through certain channels. With the increase of hospitalization days, they pay excessive attention to the negative impact of the disease on themselves, repeatedly questioning the test results. These performances are easier to capture by nurses in the form of observe-rating. The psychological states of the EW group were relatively stable, suggesting that EW can positively affect the psychological activities of patients and help them obtain an appropriate mental state. For example, a patient mentions in his writing, “I think it is a kind of success to change my mind from anger to inner peace. How important it is to experience more; even if it is a failure or danger, there is a precious treasure hidden in it. This can also be called living in abundance.” EW can allow patients to reorganize their thoughts of hospitalization and organize the event cognitively into a coherent narrative. This would allow for the event to be assimilated and, ultimately, resolved and/or forgotten, thereby alleviating the maladaptive effects of incomplete emotional processing on health.

The writing quality was highly correlated with the psychological change quantity. The length of writing and the degree of writing describing positive emotional experiences are highly correlated with the amount of mood states change in patients. This is consistent with the research of Smyth et al. (2008). The higher the information disclosure, the more benefit the individual will get. For example, one patient submitted more than 300 words each time, describing details and emotional disclosure were detailed and delicate. The main tone of the patient expression is gratitude and strength. After the intervention, the TMD score of the patient was significantly reduced. It shows that a detailed description of the event can produce a more vivid image, lead to the maximum emotional arousal of patients, and ultimately reduce their negative emotions (Holmes and Mathews, 2010). Other studies have suggested the expression of positive emotion words and negative emotion words can improve an individual’s mental state, but the effectiveness of positive emotions produces more (Burton and King, 2004; Pennebaker and Chung, 2007). The expression of positive experiences shows that patients are experiencing deliberate rumination, which is more likely to facilitate posttraumatic growth (Qu et al., 2022). Thus, the expression of positive emotion is more associated with the amount of mood change. As for the degree of writing fits the topic, it indicates that the appropriate relaxation of the topic of writing will not hinder the positive effect of writing too much, which is also consistent with the research results of Ireland et al. (2007). Although some patients did not describe a prominent topic, their focus had expanded from the current disease to other aspects. The shift of attention may play a certain role in the improvement of mental state.

Compared with other interventions, EW has the advantages of low-cost treatment and low dependence on professionals. This study showed that 77.36% of the patients believed that the EW was suitable for the patients in Fangcang hospitals. 90.57% of the patients thought that EW could regulate emotions, indicating that patients had high satisfaction with the intervention. Therefore, EW, as a novel form of psychological intervention for patients in Fangcang hospitals, has the value of promotion and use.

## Conclusion

This study contributes to help researchers and clinicians understanding the effectiveness of online EW intervention for the reduction of psychological distress among the asymptomatic COVID-19 patients in Fangcang Hospitals. The results add value to the current literature on psychological health in this specific population, and provide insights into designing larger randomized clinical trials for intervention development and implementation. However, much remains to be understood regarding EW among asymptomatic COVID-19 patients. Among four topics (emotion expression, cognitive appraisal, unlock potential, and look ahead) of the program, which topic could produce more psychological benefits? Is a whole greater than any of its parts? Further research is needed to explore the problems by conducting different experimental groups.

## Limitations

This study only explored the effect of EW on the psychological well-being of patients and did not investigate whether it could produce utility on physiologic functioning. Many studies have shown that EW can improve one’s physiologic functioning (Wilhelm and Crawford, 2020), such as improving the immune function of HIV patients (Ennis and Cartagena, 2020), etc. Future studies can verify its promoting effect on patients’ immune function and rehabilitation. In addition, the post-test of the scale was only carried out 1 day after the intervention. As some patients left the Fangcang hospitals after the intervention, a follow-up investigation could not be carried out, which could not prove the utility time of the EW. Finally, this quasi-experiment did not randomly assign the patients to the experiment and control conditions because of restrictions on the structure of Fangcang hospitals.

## Data availability statement

The raw data cannot be shared at this time as the data also forms part of an ongoing study. Requests to access the datasets should be directed to contact the corresponding author.

## Ethics statement

The studies involving human participants were reviewed and approved by the Medical Ethics Committee of the 988th Hospital. The patients/participants provided their written informed consent to participate in this study.

## Author contributions

JX, WY, and XL meet the four criteria of author guidelines. JX helped perform the analysis with constructive conclusion and clarify the confused content in original manuscript. WY and XL helped revise the introduction of the paper, polish the manuscript, and provided financial support for the project leading to this publication.

## Funding

This study received institutional support from Health Commission of Henan Province (project name: Investigation on psychological status of inpatients and research on precise intervention technology; project number LHGJ20200791).

## Conflict of interest

The authors declare that the research was conducted in the absence of any commercial or financial relationships that could be construed as a potential conflict of interest.

## Publisher’s note

All claims expressed in this article are solely those of the authors and do not necessarily represent those of their affiliated organizations, or those of the publisher, the editors and the reviewers. Any product that may be evaluated in this article, or claim that may be made by its manufacturer, is not guaranteed or endorsed by the publisher.
